# Influence of the KCNQ1 S140G Mutation on Human Ventricular Arrhythmogenesis and Pumping Performance: Simulation Study

**DOI:** 10.3389/fphys.2018.00926

**Published:** 2018-07-31

**Authors:** Da Un Jeong, Ki Moo Lim

**Affiliations:** Computational Medicine Lab, Department of IT Convergence Engineering, Kumoh National Institute of Technology, Gumi, South Korea

**Keywords:** KCNQ1 S140G mutation, ventricular arrhythmia, pumping performance, electromechanical simulation, sinus rhythm response, reentry response, dominant frequency

## Abstract

The KCNQ1 S140G mutation, which is involved in I_Ks_ current, affects atrial fibrillation. However, little is known about its effect on the mechanical behavior of the heart. Therefore, we assessed the influence of the KCNQ1 S140G mutation on ventricular electrophysiological stability and mechanical pumping performance using a multi-scale model of cardiac electromechanics. An image-based electromechanical model was used to assess the effect on electrical propagation and arrhythmogenesis of the KCNQ1 S140G mutation. In addition, it was used to compare the mechanical response under the wild-type (WT) and S140G mutation conditions. The intracellular calcium transient obtained from the electrophysiological model was applied as an input parameter to a mechanical model to implement excitation–contraction coupling. The I_Ks_ current equation was modified to account for expression of the KCNQ1 S140G mutation, and it included a scaling factor (ϕ) for mutant expressivity. The WT and S140G mutation conditions were compared at the single-cell and three-dimensional (3D) tissue levels. The action potential duration (APD) was reduced by 60% by the augmented I_Ks_ current under the S140G mutation condition, which resulted in shorter QT interval. This reduced the 3D sinus rhythm wavelength by 60% and the sustained re-entry by 56%. However, pumping efficiency of mutant ventricles was superior in sinus rhythm condition. In addition, the shortened wavelength in cardiac tissue allowed a re-entrant circuit to form and increased the probability of sustaining ventricular tachycardia and ventricular fibrillation. In contrast, under the WT condition, a normal wavelength (20.8 cm) was unlikely to initiate and sustain re-entry in the cardiac tissue. Subsequently, the S140G mutant ventricles developed a higher dominant frequency distribution range (2.0–5.3 Hz) than the WT condition (2.8–3.7 Hz). In addition, stroke volume of mutant ventricles was reduced by 65% in sustained re-entry compared to the WT condition. In conclusion, signs of the S140G mutation might be difficult to identify in sinus rhythm even though the mutant ventricles show shortened QT interval. This suggests that the KCNQ1 S140G mutation increases the risk of death by sudden cardiac arrest. In addition, the KCNQ1 S140G mutation can induce ventricular arrhythmia and lessen ventricular contractility under re-entrant conditions.

## Introduction

Atrial fibrillation is a common cardiac arrhythmia that has been the focus of recent studies aiming to reveal the correlation between atrial fibrillation and KCNQ1 S140G mutations (Kharche et al., 2012). According to animal studies and clinical data, atrial fibrillation is mainly caused by the occurrence of re-entry due to a shortened action potential duration (APD) and decreased effective refractory period (ERP) (Nattel, 2002). The S140G gain-of-function KCNQ1 mutation, which is involved in I_Ks_ channels, induces atrial fibrillation. The KCNQ1 S140G mutation in myocardial cells results in rapid development of a substantial outward K^+^ current during depolarization (Hong et al., 2005). The changes in the I_Ks_ channels caused by the KCNQ1 S140G mutation in myocardial cells increase the current through I_Ks_ channels, reducing APD and ERP, and thereby inducing atrial fibrillation (Chen et al., 2003; Kharche et al., 2012).

The electrophysiological effects of the KCNQ1 S140G mutation on myocardial cells and transgenic mouse have been investigated. Chen et al. used patch clamps and electrocardiograms (ECG) data to assess the effect of the gain-of-function S140G mutation on the KCNQ1/KCNE1 and KCNQ1/KCNE2 currents and found that this mutation caused development of atrial fibrillation by reducing APD and ERP in atrial myocytes. Therefore, the S140G mutation substantially increased the inward K^+^ current at hyperpolarization, thereby stabilizing the resting membrane potential and shortening the atrial ERP (Chen et al., 2003). Bellocq et al. reported that a gain-of-function mutation affecting I_Ks_ also results in shortening of the QT interval. Abbreviation of the action potential (AP), particularly when occurring heterogeneously, facilitates development of re-entrant arrhythmias (Bellocq et al., 2004). Hong et al. reported that I_Ks_ channels containing S140G KCNQ1 subunits were constitutively open, and exhibited instantaneous activation in response to membrane depolarization and that a gain-of-function mutation can cause atrial fibrillation and short AT syndrome (Hong et al., 2005).

The KCNQ1 S140G mutation may also affect the electrophysiology of ventricular cells. Using voltage clamps, El Harchi et al. found that the S140G mutation substantially augments the repolarizing current both early and throughout the atrial AP *in vitro* and this mutation influences ventricular AP repolarization (El Harchi et al., 2010). Furthermore, Bellocq et al. suggested that ventricular fibrillation may be induced by short QT syndrome due to the S140G mutation, which has gain-of-function characteristics (Bellocq et al., 2004). Yang et al. reported that the S140G mutation is likely responsible for atrioventricular blocks. Therefore, this mutation may be associated with other cardiac arrhythmias (Yang et al., 2007).

The above studies were performed at the cellular level, not in tissue or organs. Several computational studies have predicted the effect of mutations on cardiac tissue. Kharche et al. used computer modeling to investigate the mechanism by which the KCNQ1 S140G mutation, promotes and perpetuates atrial fibrillation. I_Ks_ was increased by the S140G mutation, which enhances atrial susceptibility to arrhythmia, facilitating initiation, and maintenance of re-entry (Kharche et al., 2012). In addition, Hancox et al. employed ventricular AP clamp experiments and ventricular APD simulation to investigate not only the mechanism by which the S140G mutation modulates the risk of atrial arrhythmia, but its effect on ventricular electrophysiology by; this mutation resulted in an abbreviated ventricular AP (Hancox et al., 2014).

These studies predicted variations in cardiac responses due to the KCNQ1 S140G mutation in atria and ventricles from an electrophysiological standpoint. However, they did not predict the effects of this mutation on cardiac mechanical responses. It is important to consider cardiac mechanical phenomena as well as electrical phenomena because cardiac mechanical contraction, which is triggered by electrical excitation, is the ultimate purpose of the heart. Also, cardiac mechanical contraction affects electrophysiological phenomena. For instance, stretch-activated channels cause changes in electrogenic pump currents or flux of specific ions through mechanical stretching of the heart (Hu and Sachs, 1997).

We recently developed a three-dimensional (3D) electromechanical model of ventricles together with a lumped parameter model of the circulatory system, and investigated ventricular electromechanical responses under various pathological conditions (Lim et al., 2012, 2013, 2015). In this study, we incorporated the KCNQ1 S140G mutation into our ventricular model to predict its effect on ventricular mechanics during the normal sinus rhythm and in the presence of re-entrant arrhythmia.

## Methods

### Model of cellular electrophysiology and cross-bridge dynamics

**Figure 1:**
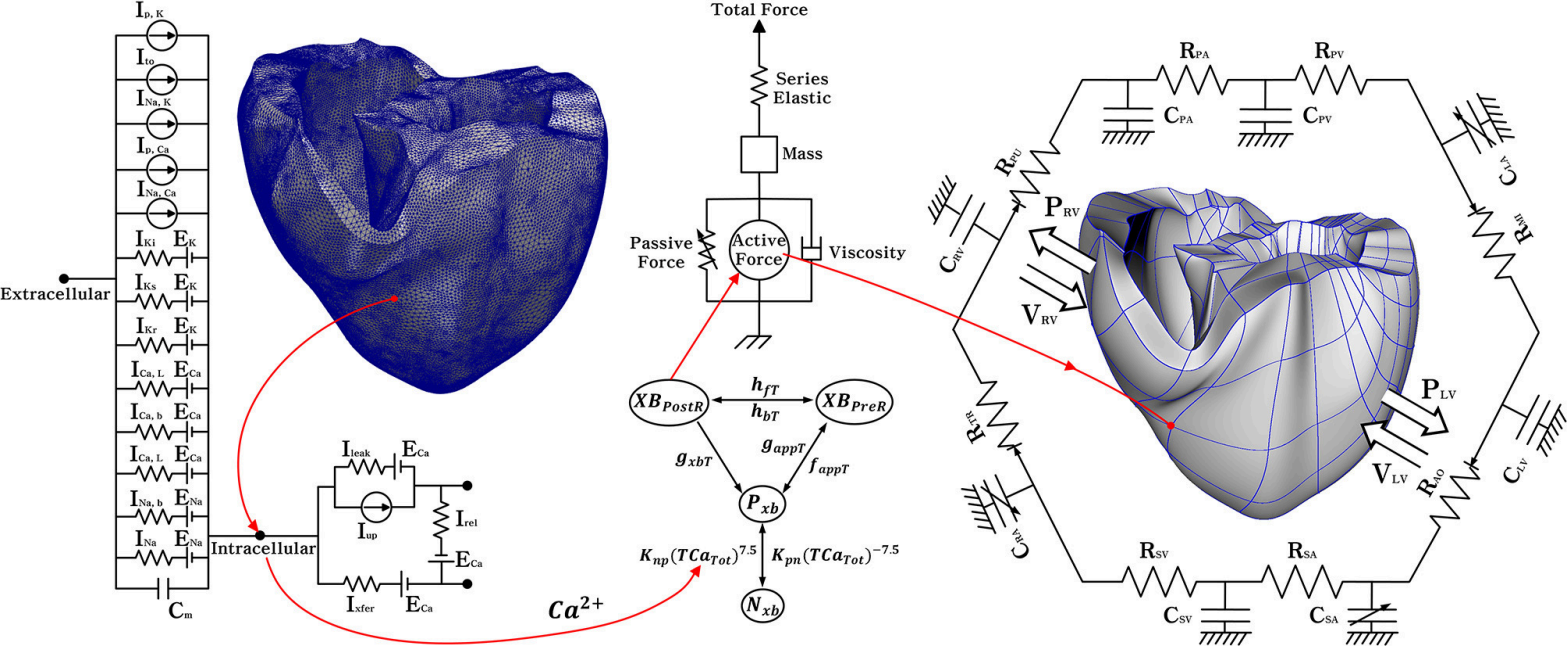
Schematic diagram of the ventricular electromechanical model. The left side of the circuit diagram is a ventricular model of electrophysiological simulation with 214,319 nodes. The electrical components of the schematic represent the current, pump, and ion exchanger from the Ten Tusscher ion model, which emulates the cell membrane for ion transport and the sarcoplasmic reticulum within cardiac cells. *I*_*p*_, _*k*_ is the current due to the K^+^ pump, *I*_*to*_ denotes the transient outward K^+^ current, *I*_*Na*_, _*K*_ is the Na^+^ −K^+^ ion exchange current, *I*_*p*_, _*Ca*_ is the current of the sarcoplasmic Ca^2+^ pump, and *I*_*Na*_, _*Ca*_ means the current mediated by the Na^+^ −Ca ^2+^ ion exchange pump. *E*_*k*_, *E*_*Ca*_, and *E*_*Na*_ are the equilibrium potentials of K^+^, Ca^2+^, and Na^+^ ions, respectively, whereas *C*_*m*_ denotes the membrane capacitance due to the phospholipid bilayer in ventricular cells. *I*_*k*1_ is the inward rectifier K_1_ current, *I*_*Ks*_ is the K^+^ current due to the slow delayed rectifier, *I*_*Ca*_, _*L*_ is the L-type inward Ca^2+^ current, and *I*_*Ca*_, _*b*_ denotes the background Ca^2+^ current. *I*_*Na*_, _*b*_ is the background Na^+^ current, and *I*_*Na*_ is the fast inward Na^+^ current. *I*_*rel*_ is the Ca^2+^ current released from the junctional sarcoplasmic reticulum (JSR), *I*_*leak*_ denotes the Ca^2+^ current that leaks from the ISR, and *I*_*up*_ is the Ca^2+^ uptake current into the network sarcoplasmic reticulum (NSR). The mechanical components on the right are the myofilament models proposed by Rice et al. *N*_*xB*_ and *P*_*xB*_ are non-permissive and permissive confirmations of regulatory proteins, and *XB*_*PreR*_ is the pre-rotated state of the myosin head in relation to binding, which contributes to stiffness but does not generate force in the absence of net motion. *XB*_*PostR*_ denotes a strongly bound myosin head and represents the isomerization that induces strain in the extensible neck region. *G*_*xbT*_ is the ATP-consuming detachment transition rate, *h*_*fT*_ and *h*_*bT*_ are the forward and backward transition rates, respectively; *f*_*aapT*_ is the cross-bridge attachment rate of transition to the first strongly-bound state *XB*_*PreR*_, and *g*_*aapT*_ is the reverse rate. *K*_*np*_ and *K*_*pn*_ are transition rates, *K*_*np*_*(TCa*_*Tot*_*)*^7.5^ is the forward rate of the nonpermissive-to-permissive transition, in the opposite direction, and *K*_*pn*_*(TCa*_*Tot*_*)*−7.5 is the backward rate of the permissive-to-nonpermissive transition. The force due to the cross-bridge can be subdivided into an active force and passive force. The active force induces the action of the cycling cross-bridge, and the passive force induces a complete muscle response with viscoelastic elements. Mass prevents instantaneous changes in muscle-shortening velocity for quick-release protocols, whereas a linear elastic element is intended to simulate the effects of the compliant end connections that take place in real muscle preparations. The model coupled with the circulatory; C_PA_, pulmonary artery compliance; R_PA_, pulmonary artery resistance; C_PV_, pulmonary vein compliance; R_PV_, pulmonary vein resistance; C_LA_, left atrium compliance; R_MI_, mitral valve resistance; C_LV_, left ventricular compliance; R_AO_, aortic valve resistance; R_SA_, systemic artery resistance; C_SA_, systemic artery compliance; R_SV_, systemic vein resistance; C_SV_, systemic vein compliance; C_RA_, right atrium compliance; R_TR_, tricuspid valve resistance; C_RV_, right ventricular compliance; R_PU_, pulmonary valve resistance; P_RV_, right ventricular pressure; V_RV_, right ventricular volume; P_LV_, left ventricular pressure; V_LV_, left ventricular volume.

To investigate the electromechanical effects of the KCNQ1 S140G mutation, we used an excitation–contraction coupling model, which is a human ventricular model with electrophysiological conduction and mechanical contraction. A model of electrophysiological conduction characteristics consisted of a circuit of concentrated circulation that simulated the mechanism of ion exchange through the plasma membrane of myocardial cells (Figure 1). We used a modified version of the validated ventricular ion model proposed by Ten Tusscher et al. (2004). To mimic the conduction of AP in myocardial cells, we applied continuum mechanics based on an electrical conduction equation (Equation 1),

(1) $$\begin{array}{r} \frac{dV_{m}}{dt}=\;-\frac{I_{ion}+I_{stim}}{C_{m}} \end{array}$$

where *V*_*m*_ is the cell membrane potential, *t* is time, *I*_*ion*_ is the sum of the transmembrane currents, *I*_*stim*_ is the current due to external stimuli, and *C*_*m*_ is the capacitance of the cell membrane.

To express the conduction phenomenon of myocardial tissue in 3D space, we combined the ordinary differential equation for electrical propagation through the ion channel with the partial differential equation for electrical stimulation in tissue (Equation 2):

$$\begin{array}{rcl} \frac{dV_{m}}{dt} & = & -\frac{I_{ion}+I_{stim}}{C_{m}}+\frac{1}{\rho_{x}SC_{m}}\frac{\partial^{2}V}{\partial^{2}x^{2}}+\frac{1}{\rho_{y}SC_{m}}\frac{\partial^{2}V}{\partial^{2}y^{2}} \end{array}$$

(2) $$\begin{array}{r} +\frac{1}{\rho_{z}SC_{m}}\frac{\partial^{2}V}{\partial^{2}z^{2}} \end{array}$$

where ρ_*x*_, ρ_*y*_, and ρ_*z*_ represent the cell resistance in x, y, and z directions, and *S* represent the ratio of the volume to the surface. Following Ten Tusscher et al., the sum of the transmembrane currents, *I*_*ion*_, was calculated by Equation (3):

$$\begin{array}{rcl} I_{ion} & = & I_{Na}+I_{K1}+I_{to}+I_{Kr}+{I^{\prime }}_{Ks}+I_{Ca,L}+I_{Na,\;Ca}+I_{Na,K} \end{array}$$

(3) $$\begin{array}{r} +I_{p,Ca}+I_{p,K}+I_{b,Ca}+I_{b,Na} \end{array}$$

where *I*_*Na*_ represents the Na^+^ current, *I*_*K*1_, *I*_*to*_, and *I*_*Kr*_ are the K^+^ currents, namely the inward rectifier K^+^ current, transient outward K^+^ current, and rapid delayed rectifier K^+^ current, respectively. *I'*_*Ks*_ is the slow delayed rectifier K^+^ current due to KCNQ1 S140G mutation. This is explained in detail in the following paragraphs. *I*_*Ca*_, _*L*_ is the L-type inward Ca^2+^ current, *I*_*Na*_, _*Ca*_ is the Na^+^-Ca^2+^ exchange current, *I*_*Na*_, _*k*_ denotes the Na^+^-K^+^ exchange current, *I*_*p*_, _*Ca*_ is the current of the Ca^2+^ pump, *I*_*p*_, _*K*_ is the current of the K^+^ pump, *I*_*b*_, _*Ca*_ is the background Ca^2+^ current, and *I*_*b*_, _*Na*_ represents the background Na^+^ current.

To confirm the electrophysiological changes in ventricular cells caused by the KCNQ1 S140G mutation, we used the *I*_*Ks*_ current equation (Equation 4) proposed by the Ten Tusscher ion model and the mutant *I*_*Ks*_ current equation (Equation 5) of Kharche et al. (Ten Tusscher et al., 2004; Kharche et al., 2012):

(4) $$\begin{array}{r} {I^{\prime }}_{Ks}=\;I_{Ks}+\varphi G_{Ks}\left(V-{E^{{}^{\prime }}}_{rev}\right) \end{array}$$

(5) $$\begin{array}{r} I_{Ks}=G_{Ks}x_{s}^{2}\left(V-E_{Ks}\right) \end{array}$$

where *G*_*Ks*_ denotes the conductance of the K^+^ ion channel, which was set to 0.392·1.3 mS/μF in this study. *E'*_*rev*_ is the mutant reversal potential due to the instantaneous component of the K^+^ ion channel, which was set at −75.3 mV. ϕ is the scaling factor for the S140G mutation expressivity, which was set to 0.1. This is the value used for observing the state of the ventricles, which is intermediate mutation. This value was established based on clinical studies that showed rapid changes in protein channels depending on the expression levels of the S140G mutation (Kharche et al., 2012). *x*_*s*_ is the activated gate variable, which was set to 0.00357 to represent K^+^ channels in a steady state. *E*_*Ks*_ is the equilibrium potential of the K^+^ channel, in which the initial value was set to zero (Ten Tusscher et al., 2004; Kharche et al., 2012). In particular, we used transient calcium information of electrophysiological simulation as an input for excitation–contraction coupling. Therefore, we applied the calcium dynamics equation of the Ten Tusscher ion model to induce contraction of the myofilaments and generate tension through the Ca^2+^-induced Ca^2+^-released (CICR) current.

(6) $$\begin{array}{rcl} I_{leak} & = & V_{leak}\left(Ca_{sr}-Ca_{i}\right) \end{array}$$

(7) $$\begin{array}{rcl} I_{up} & = & \frac{V_{maxup}}{1+K_{up}^{2}/Ca_{i}^{2}} \end{array}$$

(8) $$\begin{array}{rcl} I_{rel} & = & \left(a_{rel}\frac{Ca_{sr}^{2}}{b_{rel}^{2}+Ca_{sr}^{2}}+c_{rel}\right)dg \end{array}$$

$$\begin{array}{rcl} \frac{dCa_{itotal}}{dt} & = & -\frac{I_{Ca,L}+I_{b,Ca}+I_{p,Ca}-2I_{Na,Ca}}{2V_{C}F} \end{array}$$

(9) $$\begin{array}{r} +I_{leak}-I_{up}+I_{rel} \end{array}$$

(10) $$\begin{array}{rcl} \frac{dCa_{srtotal}}{dt} & = & \frac{V_{c}}{V_{SR}}\left(-I_{leak}+I_{up}-I_{rel}\right) \end{array}$$

where *I*_*leak*_, *I*_*up*_, and *I*_*rel*_ denote leakage current from sarcoplasmic reticulum to cytoplasm, pump current taking up calcium in sarcoplasmic reticulum, and CICR current, respectively. *V*_*leak*_ is maximal *I*_*leak*_, *V*_*maxup*_ is maximal *I*_*up*_. *Ca*_*i*_ and *Ca*_*sr*_ are for the free calcium concentration in cytoplasm and in sarcoplasmic reticulum. *K*_*up*_ is half-saturation constant of *I*_*up*_. *a*_*rel*_, *b*_*rel*_, and *c*_*rel*_ are maximal *Ca*_*sr*_-dependent *I*_*rel*_, *Ca*_*sr*_ half-saturation constant of *I*_*rel*_, and maximal *Ca*_*sr*_-independent *I*_*rel*_, respectively. *d* is the activation gate of *I*_*rel*_, and *g* is the calcium-dependent inactivation gate of *I*_*rel*_. *Ca*_*itotal*_ refers to the amount of Ca^2+^ in the cytoplasm and *Ca*_*srtotal*_ denotes the total amount of Ca^2+^ in the sarcoplasmic reticulum. *V*_*c*_ and *V*_*SR*_ are the volume of cytoplasmic and sarcoplasmic reticulum, respectively. *F* is Faraday constant.

In addition, to mimic cardiac muscle contraction, we referenced a mechanical cross-bridge cycling model of the myofilament, suggested by Rice et al. (2008). To express myofilament contraction, we used the equation of normalized active force (Equation 11),

(11) $$\begin{array}{r} F_{active}\left(x\right)=SOVF_{thick}\left(x\right)\times \frac{xXB_{PreR}\times XB_{PreR}+xXB_{PostR}\times XB_{PostR}}{x_{0}\times XB_{PostR}^{Max}} \end{array}$$

where SOVF_thick_ is the single-overlap function for the thick filament, XB_PreR_ is state of cross-bridge pre-rotated, XB_PostR_ is state of cross-bridge post-rotated, which represents the isomerization to induce strain in the extensible neck region, xXB_PreR_ is the average distortion of XB_PreR_, xXB_PostR_ is the average distortion of XB_PostR_, $XB_{PostR}^{Max}$ is scaling factors for state occupancy computed under optimal conditions, x_0_ is cross-bridge distortion length.

### Three-dimensional ventricular electromechanical models

In this study, we used the 3D human ventricular finite element model, which has a concentrated physiological circulatory system based on a Windkessel element. The electrophysiological simulation model consisted of 214,319 finite elements of a tetrahedral structure. The model for mechanical contraction simulation consists of 14,720 finite elements, based on Hermite, to represent the natural 3D curve of the heart surface.

For 3D electrophysiology simulation under sinus rhythm conditions, we used the ventricular model with Purkinje fibers mesh, which are distributed on the sub-endocardial surface. This model mimics conduction of electrical simulation from the AV node through the ends of the Purkinje fibers to the entire ventricle (Berenfeld and Jalife, 1998). In addition, considering differences in ventricle structural characteristics and thickness, we assumed that the ventricle tissue was heterogeneous, and that the conductance varied among the parts of the ventricle; namely, endocardium, mid-myocardium, and epicardium. A detailed description of each conductance value can be found in the simulation protocols section. For distinguish each parts of the ventricle, we applied electrical stimulation to the sub-endocardial surface and the endocardial surface, respectively. The tissues that propagated for 2 ms were determined to be endocardium and epicardium. In addition, we determined the mid-myocardium as the middle tissue, which was neither endocardium nor epicardium.

Mathematical description of mechanical contraction in cardiac tissue is based on continuum mechanics, where it is assumed for myocardium to be hyper-elastic and nearly incompressible material, and to have the passive mechanical properties defined by an exponential strain (W) function (Guccione et al., 1995; Usyk et al., 2002).

(12) $$\begin{array}{rcl} W & = & \frac{C}{2}\left(e^{Q}-1\right) \end{array}$$

(13) $$\begin{array}{rcl} Q & = & b_{1}E_{ff}^{2}\pm b_{2}\left(E_{rr}^{2}+E_{cc}^{2}+{2E}_{rc}^{2}\right)+{2b}_{3}\left(E_{fr}^{2}+E_{fc}^{2}\right) \end{array}$$

(14) $$\begin{array}{rcl} E_{\alpha \beta } & = & \frac{1}{2}\left(\frac{{\partial x}^{k}}{{\partial v}^{\alpha }}\frac{{\partial x}^{k}}{{\partial v}^{\beta }}-\delta_{\alpha \beta }\right) \end{array}$$

where C is the material constants and set to 2 kPa, b_1_ is 8, b_2_ is 2, and b_3_ is 4, which are determined with the orthotropic electrical conductivity and passive mechanical properties of the myocardium by the laminar sheet-norminal direction and fiber orientation information (For more information, see Supplementary Material). The Langian Green's strains *E*_αβ_ are referred to the local fiber coordinate system. *x*^*k*^ is transformed rectangular Cartesian coordinates, and δ_αβ_ is the Kronecker delta.

For simulate hemodynamic responses, the interactions between the blood and ventricles, the finite element electromechanical model of the human ventricle was coupled with a circulatory model using coupling method of Gurev et al. (2011) as in Figure 1.The circulatory model used in this study was developed based on cardiovascular model of Kerckhoffs et al. (2007). Therefore, it can derive the blood characterized by pressure, volume, and flow of ventricles. In particular, the ventricular pressure expressed by the following time-varying mathematical equation.

(15) $$\begin{array}{rcl} Pressure & = & \;C^{-1}\left(t\right)\left(V-V_{rest}\left(t\right)\right) \end{array}$$

(16) $$\begin{array}{rcl} C & = & \;y_{v}\left(C_{\max}-C_{\min}\right)+\;C_{\min} \end{array}$$

where *C* is time-varying compliance matrix. *C*_*max*_ and *C*_*min*_ are compliance for ventricular fully activate state and passive state, respectively. *y*_*v*_ denotes ventricular active function. *V* is ventricular volume and *V*_*rest*_ is ventricular volume at zero pressure.

(17) $$\begin{array}{rcl} V_{LV} & = & \;-R_{LA}{\overset{{}^{\circ}}{Q}}_{LA}+\;\frac{1}{C_{LA}}Q_{LA} \end{array}$$

(18) $$V_{rest}\;={\left(1-y_{v}\right)}^{*}\left[\begin{array}{c} V_{L,rest,d}\;V_{L,rest,s}\\ V_{R,rest,d}\;V_{R,rest,s} \end{array}\right]+\left[\begin{array}{c} V_{L,rest,s}\\ V_{R,rest,d} \end{array}\right]$$

where *V*_*L, rest, d*_, *V*_*L, rest, s*_, *V*_*R, rest, d*_, and *V*_*R, rest, s*_ are diastolic volume and systolic volume of the left and right ventricle, respectively.

### Simulation protocols

First, we investigated the effect of the KCNQ1 S140G mutation on cellular electrophysiology and the APD restitution curve. Second, we performed 3D simulation to assess the effect of the S140G mutation on the sinus rhythm response. Third, we evaluated induction of arrhythmogenesis due to the KCNQ1 S140G mutation. Fourth, we compared cardiac pumping efficiency under the WT and S140G mutation conditions with sustained re-entry.

In the cellular electrophysiological simulation, we compared the I_Ks_ current and APD_90_ between the S140G mutation and WT conditions. In addition, using a dynamic restitution protocol, we compared the action potential duration-basic cycle length (APD-BCL) curves and action potential duration-diastolic interval (APD-DI) curves; the BCL was 1,000 and 20 ms under the S140G mutation and WT conditions, respectively. In addition, we investigated the electrical phenomena caused by the S140G mutation in the ventricular endocardium, midmyocardium, and epicardium cells. For this purpose, we applied electrical conductivity as follows: for the endocardium, G_ks_ = 0.392^*^1.3 mS/μF and G_to_ = 0.073 mS/μF; for the midmyocardium, G_ks_ = 0.098^*^2.0 mS/μF and G_to_ = 0.294 mS/μF; for the epicardium, G_ks_ = 0.392^*^1.3 mS/μF and G_to_ = 0.294 mS/μF. These conductance values were derived from the human ventricular model of Ten Tusscher et al., which is a validated ventricular model (Ten Tusscher et al., 2004).

In the 3D simulation, we compared the cardiac pumping efficiency of the sinus rhythm under the WT and S140G mutation conditions. We first performed a 3D electrophysiological simulation and extracted the transient Ca^2+^ information. To mimic cardiac excitation–contraction coupling, we used the Ca^2+^ data, which is extracted from electrophysiological simulation, as an input in the mechanical contraction simulation. We set the BCL for sinus rhythm to 600 ms. To evaluate the mechanical behavior of ventricular tissue in steady state, we used only the result of the last cycle of the electrophysiological simulation (12 s).

We introduced the concepts of electrical activation time (EAT) and electrical deactivation time (EDT) to quantitatively confirm the electrophysiological changes caused by the KCNQ1 S140G mutation. EAT is defined as the time at which depolarization begins in ventricle myocardial cells. In this study, EAT was the time at which the AP reached −30 mV. EDT was defined as the time at which repolarization was terminated in ventricle myocardial cells. Here, EDT was the time at which the AP reached −75 mV.

To quantitatively assess the effect on pumping efficiency of the changes in I_Ks_ channels and AP caused by the S140G mutation, we used the following mechanical analysis methods: pressure–volume loop (PV loop), stroke volume, ejection fraction, and ATP consumption.

Finally, we evaluated the effect of the S140G mutation on the occurrence of ventricular fibrillation by conducting a re-entry generation simulation. We performed a sustained re-entry response simulation to compare the mechanical contraction efficiency when the re-entry wave reached steady state under the WT and S140G mutation conditions. In the re-entry generation simulation, re-entry was generated at a conduction velocity of 65 cm/s under normal conditions using S1–S2 protocols. In S1–S2 protocols, the S1 stimulus was applied three times at 600 ms intervals, and the S2 stimulus was applied when the tail of the third wave reached the middle of the ventricle. We compared arrhythmogenesis and maintenance of re-entry between the S140g mutation and WT conditions.

To generate re-entrant wave in the sustained re-entry simulation, the S1–S2 protocol was applied with a very low conduction velocity (20 cm/s). When re-entry was generated under the S140G mutation and WT conditions, we saved all dependent variables of the cellular state at the moment at which the re-entry waves were maintained. These saved cellular state variables were used as the initial values in the next simulation, which involved a normal conduction velocity (70 cm/s).

We calculated the dominant frequency of each node in the ventricular model to quantitatively compare the electrophysiological changes due to the KCNQ1 S140G mutation during re-entry. The dominant frequency was determined using the fast Fourier transform (FFT) function of MATLAB, with the sampling rate set to 0.01. The dominant frequency is defined as the frequency corresponding to the maximum power calculated by FFT. Additionally, to quantitatively assess cardiac mechanical behavior according to the electrophysiological changes caused by the S140G mutation during sustained re-entry, we evaluated ventricular pressure, volume, and ATP consumption rate.

## Results

### Effect on electrophysiological activity of the S140G mutation

**Figure 2:**
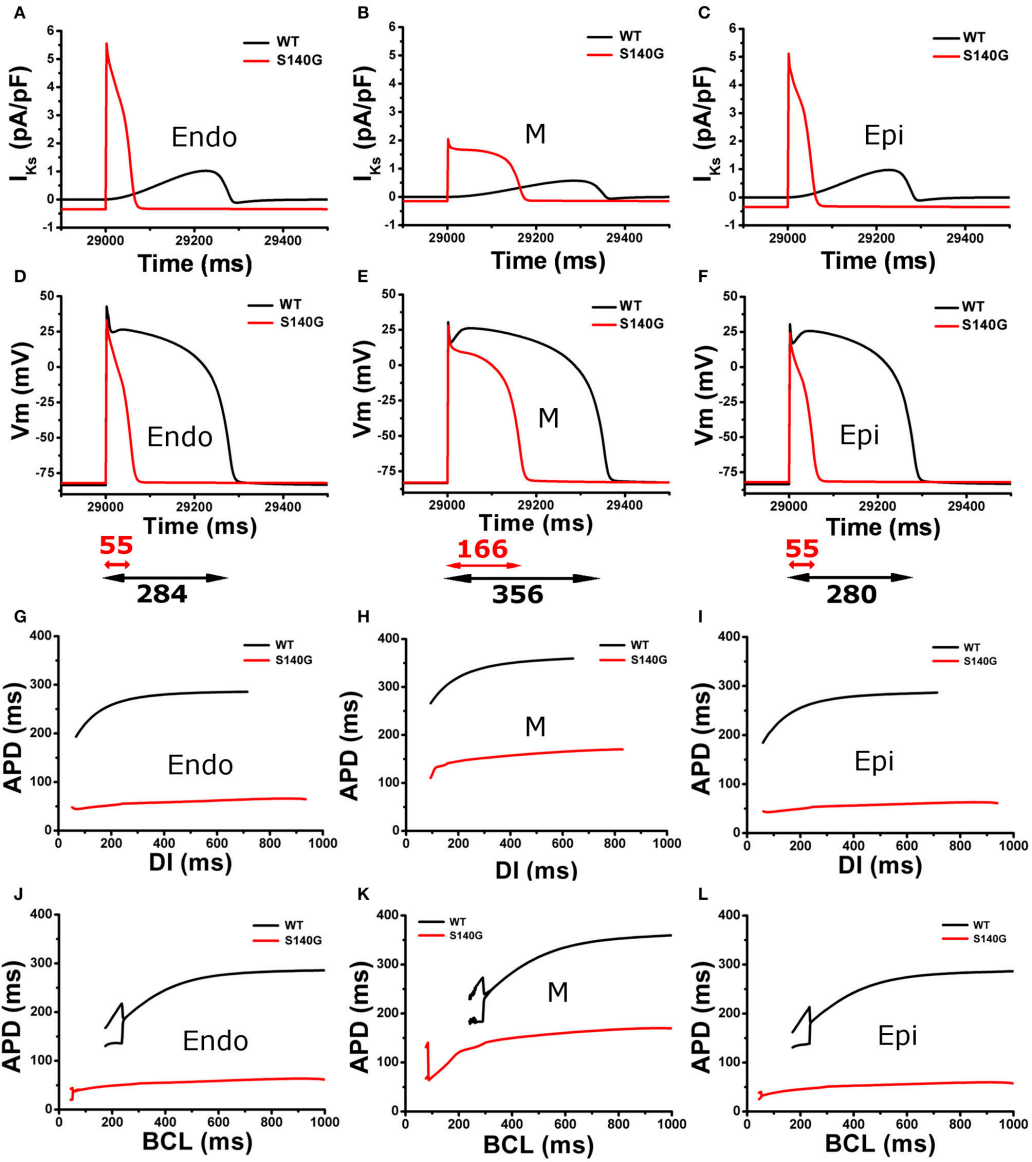
Single-cell simulations using various myocardial cell types under the WT and S140G mutation conditions. I_Ks_ currents **(A–C)**, the corresponding AP shapes **(D–F)**, and APDR curves **(G–L)** under the WT and KCNQ1 S140G mutation conditions in ventricular endocardium (Endo), mid-myocardium (M), and epicardium (Epi) cells. APD, action potential duration; DI, diastolic interval and; BCL, basic cycle length.

We assessed the effect of the KCNQ1 S140G mutation on I_Ks_ current and the effect of the variation in I_Ks_ on the shape of the AP. Under the S140G mutation condition, I_Ks_ currents were significantly higher in the early phase of the pacing cycle compared to under the WT condition in endocardial, mid-myocardial, and epicardial cells (Figures 2A–C). However, I_Ks_ channels under the S140G mutation condition closed more rapidly than under the WT condition, which resulted in early termination of I_Ks_ currents. In endocardial cells, I_Ks_ currents were sustained for 284 ms under the WT condition (average current 0.27 mA/pF) and 80 ms under the S140G mutation condition (0.64 mA/pF). In mid-myocardial cells, I_Ks_ currents were sustained for 332 ms under the WT condition (average current 0.19 mA/pF) and 180 ms under the S140G mutation condition (0.50 mA/pF). In epicardial cells, I_Ks_ currents were sustained for 284 ms under the WT condition (average current 0.25 mA/pF) and 76 ms under the S140G mutation condition (60 mA/pF).

Due to these changes in I_Ks_ currents, the APD_90_ under the S140G mutation condition was significantly reduced compared to that under the WT condition. In endocardial cells, the APD_90_ was 55 ms under the S140G mutation condition, but 284 ms under the WT condition. In mid-myocardial cells, the APD_90_ was 166 ms under the S140G mutation condition, but 365 ms under the WT condition. In epicardial cells, the APD_90_ was 55 ms under the S140G mutation condition, but 280 ms under the WT condition (Figures 2D–F).

To observe the rate dependency of APD and wave-front stability, APD restitution curves were generated using the dynamic restitution protocol under WT and S140G mutation conditions in three cell types. The APD restitution curves under the WT condition matched the results reported by Ten Tusscher et al. (2004). The maximum slope of the APD restitution curve was >1 and < 1 under the WT and S140G mutation conditions, respectively (Figures 2J–L). According to Ten Tusscher et al., cells under the S140G mutation condition, which have maximum APD restitution curve slopes of < 1, experience the alternans phenomenon at lower BCL values than they do under the WT condition (Ten Tusscher and Panfilov, 2006). Alternans under the S140G mutation condition occurred at 50 ms in the endocardium, 100 ms in the mid-myocardium, and 50 ms in the epicardium; in contrast, they occurred at 244 ms in the endocardium, 310 ms in the mid-myocardium, and 235 ms in the epicardium under the WT condition.

### 3D electromechanical simulation

The changes in electrophysiological activity due to the KCNQ1 S140G mutation described above were detected in single-cellular levels. We thus investigated the effect of these electrophysiological variations on cardiac mechanical behavior using an image-based 3D ventricular electromechanical model.

#### 3D sinus rhythm

**Figure 3:**
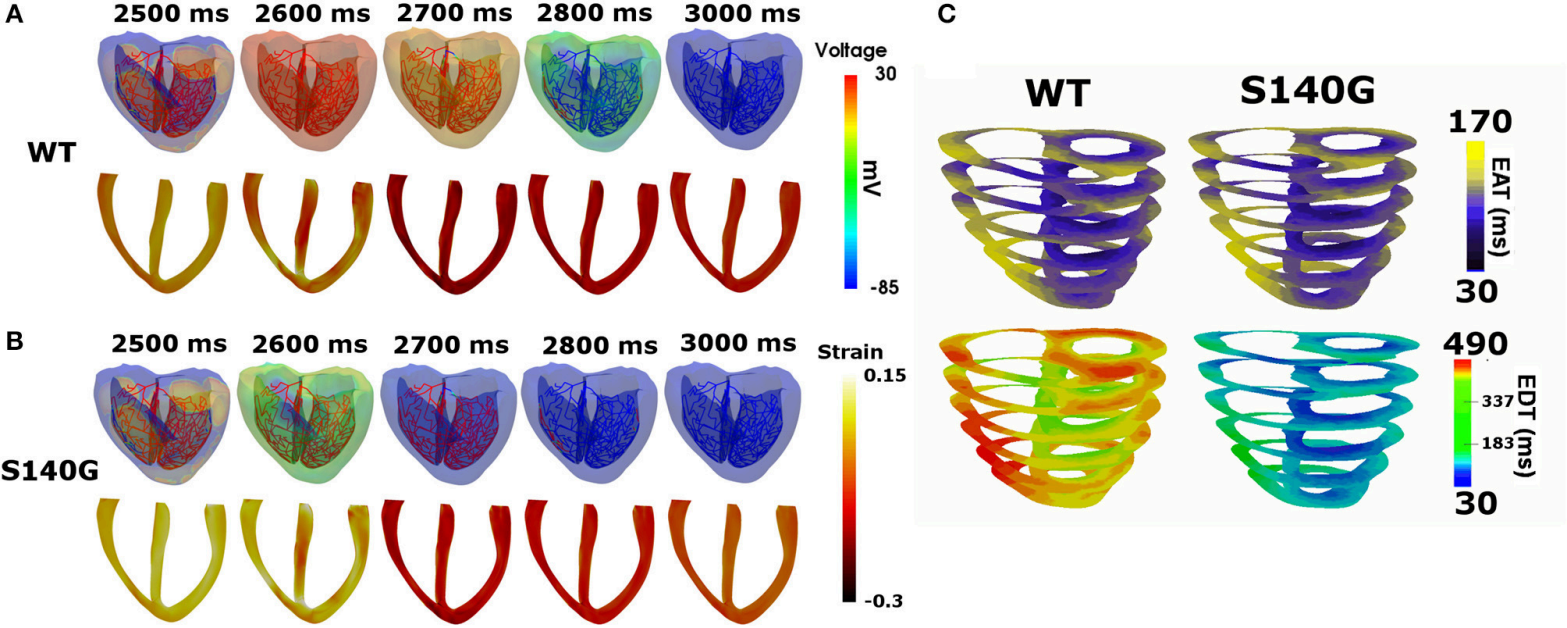
Sinus rhythm response in the 3D ventricular tissue model under the WT and S140G mutation conditions. Snapshots of the transmural distribution of membrane potential (top), strain (bottom) **(A,B)** and electrical activation time (EAT) and electrical deactivation time (EDT) during one cycle of sinus pacing under the WT and KCNQ1 S140G mutation condition **(C)**. The anisotropy ratio of CV (longitudinal to transverse) is 1.5.

It is assumed that in sinus rhythm, electrical impulse propagates from the AV node through the Purkinje fiber mesh. Because the conduction velocity through the Purkinje fibers is identical under the S140G mutation and WT conditions (100 cm/s), electrical depolarization of cardiac muscle from the Purkinje terminal node was triggered at the same time. Although electrical depolarization was applied simultaneously under the WT and S140G mutation conditions, electrical repolarization spread through the ventricular tissue at different velocities (Figures 3A,B). The transmural distribution of membrane potential also differed between the two conditions due to the differently shaped action potentials. APD_90_ was 309 ms under the WT condition compared to 126 ms under the S140G mutation condition. The conduction velocity from the apex to the top of the ventricles was 67.4 and 64.7 cm/s under the WT and S140G mutant conditions, respectively. Accordingly, the conduction wavelength was 20.8 cm under the WT and 8.2 cm under the S140G mutation condition.

Figure 3C shows the transmural distribution of the EAT and EDT under the WT and S140G mutation condition. The EAT was similar, but the EDT differed significantly between the WT and S140G mutation conditions. The EDT was 337–470 ms under the WT and 30–183 ms under the S140G mutation condition. The difference between the maximum and minimum EAT, which reflects the QRS width in ECG, was 140 ms under both conditions. The difference between the maximum EDT and minimum EAT, which reflects the QT interval was 460 and 290 ms under the WT and S140G mutation conditions, respectively.

**Figure 4:**
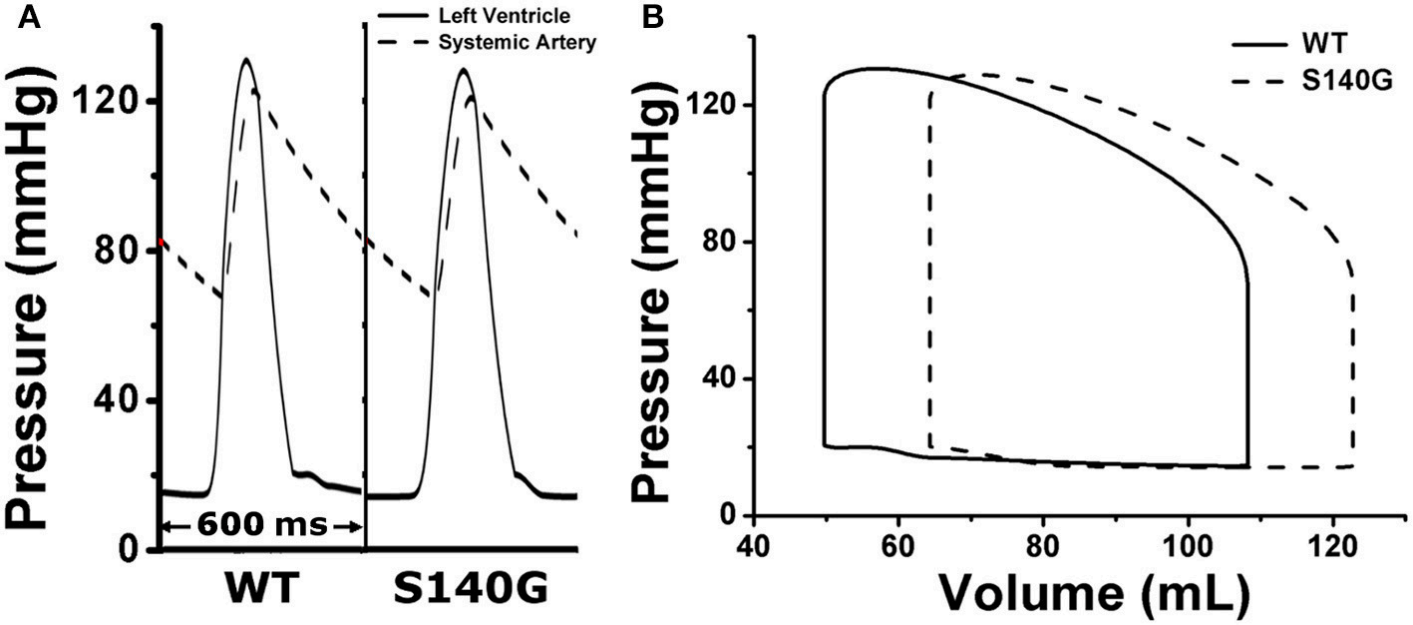
Pressure in the left ventricle and systemic artery **(A)** and the pressure–volume (PV) loop of the left ventricle **(B)** under the WT and S140G mutation conditions.

We assessed the effect of the electrophysiological changes caused by the KCNQ1 S140G mutation on ventricular mechanical contraction in sinus rhythm (Figure 4). During sinus rhythm, the pressure in the left ventricle (LV) and the systemic artery was slightly decreased under the S140G mutation condition (Figure 4A). The pressure–volume loop of the LV was shifted to the right, which resulted in an increased LV end-diastolic volume under the S140G mutation condition (Figure 4B). Therefore, the LV ejection fraction under the S140G mutation condition was significantly smaller than that under the WT condition (47.6 and 54.2%, respectively), despite the slight difference in LV stroke volumes (58.6 and 58.3 mL under the WT and S140G mutation conditions, respectively) (Table 1).

**Table 1:** Ventricular mechanical responses under the WT and S140G mutation conditions.

	**Sinus rhythm response**		**Re-entry response**	
	**WT**	**S140G**	**WT**	**S140G**
Stroke volume (mL)	58.6	58.3	3.2	1.1
Ejection fraction (%)	54.2	47.6		
Stroke work (mm Hg·mL)	5,768	5,685		
Contractile ATP consumption rate (s^−1^)	47.6	36.0		
Stroke work/Contractile ATP consumption (mm Hg·mL/BCL)	121.2	157.9		

*WT, wild type condition; S140G, KCNQ1 S140G mutation condition*.

Under the S140G mutation condition, ventricles did less stroke work (5,685 and 5,768 mm Hg·mL under the S140G mutation and WT conditions) and consumed less contractile ATP (36 /BCL in S140G and 47.6 /BCL in WT) than under the WT condition. Ventricular pumping efficacy, i.e., LV stroke work divided by contractile ATP consumption, was 157.9 and 121.2 mm Hg·mL/BCL under the S140G mutation and WT conditions, respectively.

#### 3D re-entrant dynamics

**Figure 5:**
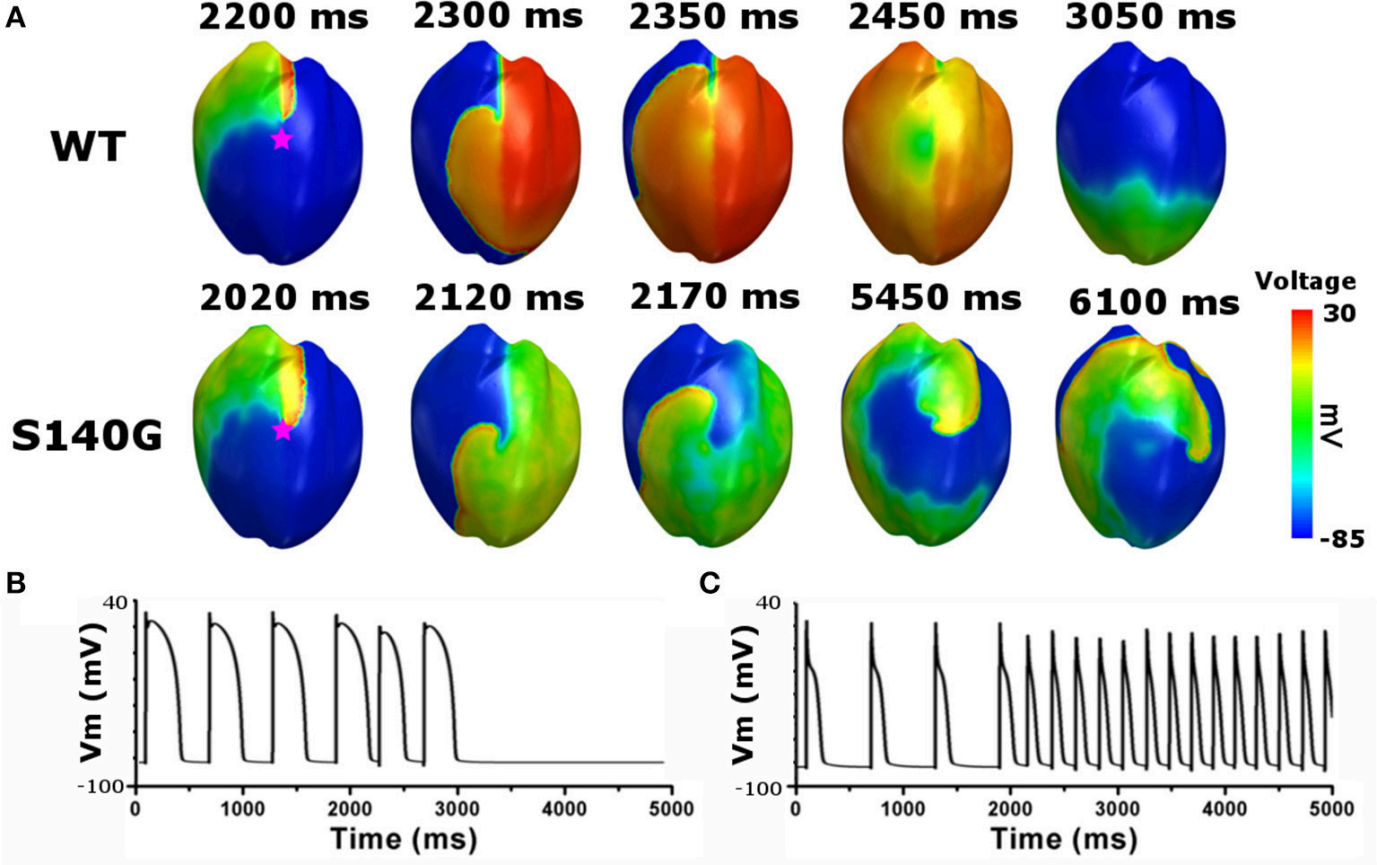
Membrane distribution of 3D electrical re-entry generation simulation under the WT and S140G mutation conditions. Snapshots of the transmural distribution of membrane potential over time **(A)**, and AP shapes for re-entry generation under the WT **(B)**, and S140G mutation **(C)** conditions. AP shapes were obtained at the points marked by red stars.

To investigate the effect of the KCNQ1 S140G mutation on ventricular arrhythmogenesis, we performed a re-entrant wave dynamics simulation using an image-based 3D ventricle model under the WT and S140G mutation conditions. Under the S140G mutation condition, a re-entrant wave was generated with the S1–S2 protocol and sustained re-entry occurred at a 65 cm/s conduction velocity and 5 Hz rotation rate (lower panel in Figure 5A). Under the WT condition, no re-entrant wave was generated, and wave propagation terminated at 3.050 ms (upper panel in Figure 5A) with identical tissue conductivity (65 cm/s). Spikes of 5 Hz in the action potential began at 2 s under the S140G mutation condition (Figure 5C). whereas no action potential was evident after 3 s under the WT condition (Figure 5B).

**Figure 6:**
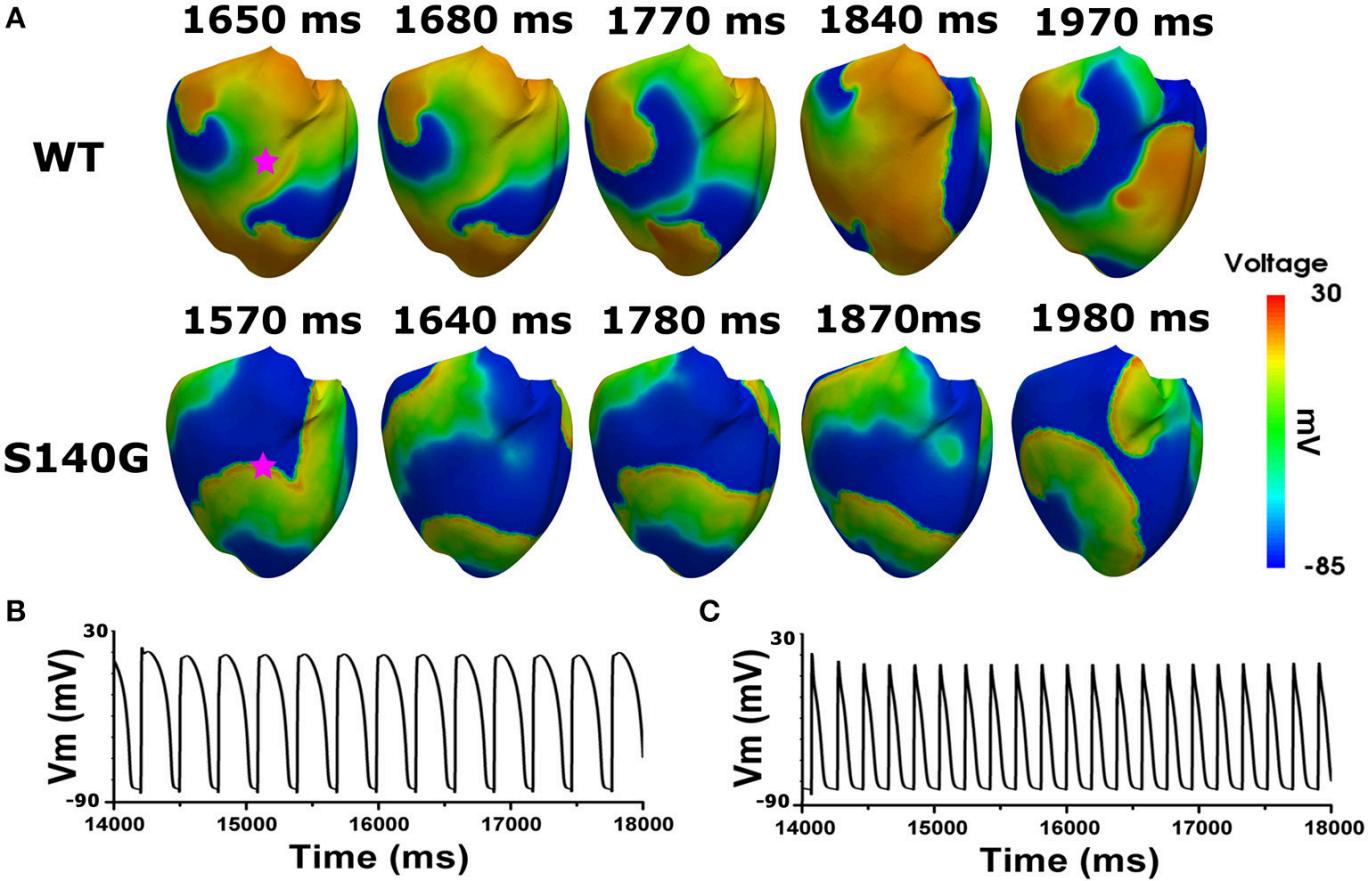
Membrane distribution of 3D electrical simulation during sustained re-entry under the WT and S140G mutation conditions. Snapshots of the transmural distribution of membrane potential over time during re-entry **(A)**, and AP shapes during re-entry under the WT **(B)**, and S140G **(C)** conditions. AP shapes were obtained at the points marked by red stars.

Figure 6 shows the electrical wave propagation patterns under the WT and S140G mutation conditions with sustained re-entry. In epicardial cells, the estimated average APD was 106 and 240 ms under the S140G mutation and WT conditions, respectively. The estimated wavelength was 7 and 16 cm under the S140G mutation and WT conditions, respectively. Therefore, the rotation rate was significantly higher under the S140G mutation than under the WT conditions (4.9 and 3 Hz, respectively).

**Figure 7:**
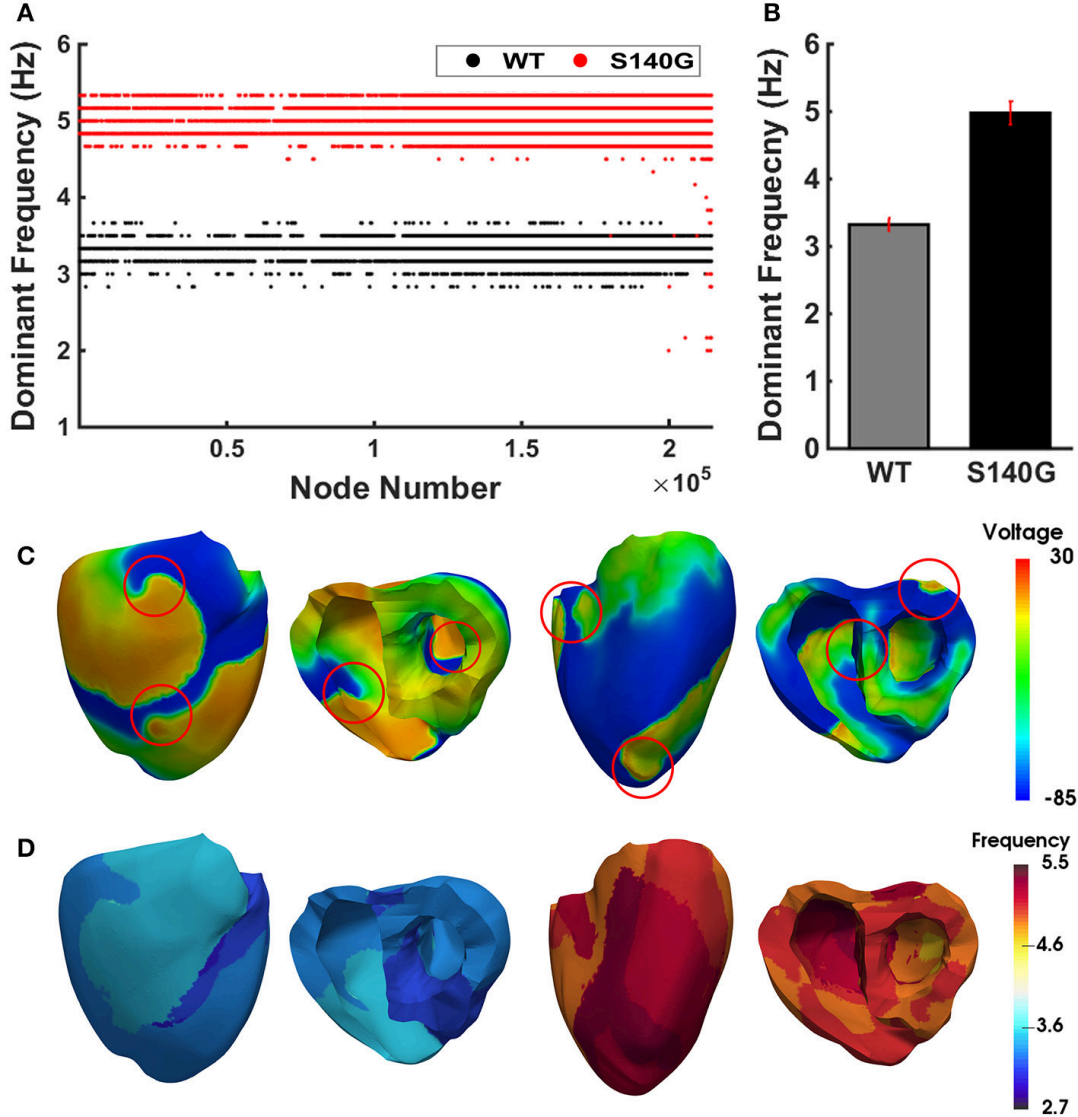
The re-entrant dynamics response under the WT and S140G mutation conditions. Dominant frequency **(A)** of each node in the 3D ventricle model and frequency variance **(B)** under the WT and S140G mutation conditions. Contour of the transmural distribution of re-entrant dynamic wave in the 3D ventricular model under the WT and S140G mutation conditions **(C)**. Contour of the dominant frequency in each node under the WT and S140G mutation conditions **(D)**.

Figure 7 shows the dominant frequency of each node during re-entry under the WT and S140G mutation conditions. The distribution of the dominant frequency under the WT condition was 2.8 to 3.7 Hz (0.9 Hz bandwidth). However, the distribution of the dominant frequency under the S140G mutation condition was 2.0–5.3 Hz (3.3 Hz bandwidth). Therefore, the dominant frequency of all nodes was more widely distributed under the S140G mutant condition. The average dominant frequency was 3.3 and 5.0 Hz under the WT and S140G mutation conditions, respectively. The lowest dominant frequency under the WT condition was 2.875 Hz, compared to 2.0 Hz under the S140G mutation condition. The probability of alternans was lower in the lower than in the higher range of the dominant frequency under the WT and S140G mutation conditions.

**Figure 8:**
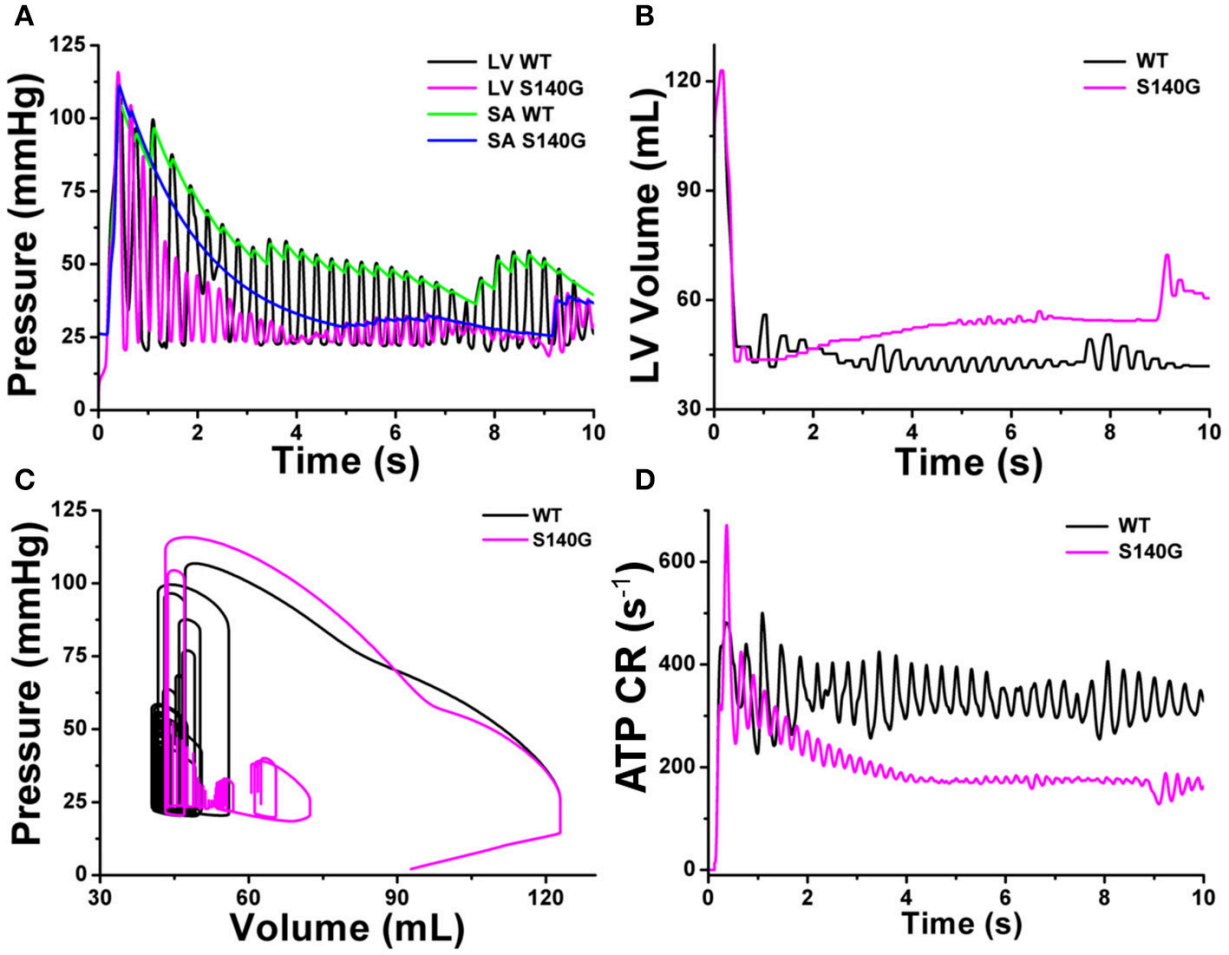
Cardiac mechanical response of a 3D re-entrant dynamics simulation under the WT and S140G mutation conditions. Pressure in the left ventricle and aorta **(A)**, volume of the left ventricle **(B)**, PV loop **(C)**, and ATP CR (consumption rate) **(D)** under the WT and KCNQ1 S140G mutation conditions.

Figure 8 shows the mechanical responses, which are coupled to the electrical responses (Figure 6). The rate of fluctuation in pressure in the LV and aorta were identical to the rotation rate of re-entry in Figure 6. LV peak pressure and average aortic pressure were lower under the S140G mutation condition than under the WT condition (Figure 8A). Variation in LV volume according to the rate of fluctuation in LV pressure was observed under the WT condition, but was less clear under the S140G mutation condition (Figure 8B). Accordingly, the average stroke volume during the meaningful period (2.778–7.254 ms) under the WT condition was 3.2 mL, while that from 4.970 to 6.924 ms under the S140G mutation condition was 1.1 mL (Table 1). Here, “meaning period” indicates the period during which blood is pumped in and out of the ventricle. Accordingly, the LV pressure–volume loop under the S140G mutation condition was shifted to the right, and the area of the loop was smaller than under the WT condition (Figure 8C). In addition, during re-entry, the contractile ATP consumption rate was lower under the S140G mutation condition than under the WT condition (Figure 8D).

## Discussion

We evaluated the effect of the KCNQ1 S140G mutation on ventricular arrhythmogenesis and mechanical behavior during normal sinus rhythm and re-entrant arrhythmia using an image-based finite element electromechanical model. This is the first study of cardiac electrophysiology and mechanics in the presence of the KCNQ1 S140G mutation. The main findings of this study were as follows:

1. In a cellular electrophysiology simulation, the KCNQ1 S140G mutation increased the I_Ks_ current density and reduced the APD in ventricular endocardium, mid-myocardium, and epicardium cells (Figure 2).
2. In sinus rhythm, ventricles repolarized more rapidly under the KCNQ1 S140G mutation condition, which decreased the EDT distribution range compared to the WT condition (Figure 3).
3. Mutant ventricles exhibited similar stroke volumes and stroke work levels under the KCNQ1 S140G mutation and WT conditions, but consumed significantly less contractile ATP under the former condition (Figure 4 and Table 1). Thus, pumping efficiency was superior under the KCNQ1 S140G mutation condition.
4. In the presence of a normal conduction velocity (70 cm/s), only mutant ventricles generated and maintained a stable re-entrant wave (Figure 5). This observation is consistent with the findings of Kharche et al.
5. In 3D simulation, spiral break-up occurred under both the WT and S140G mutation conditions (Figure 6). The conduction wavelength was reduced under the KCNQ1 S140G mutation condition. Ventricles developed a higher dominant frequency distribution range (4–5.5 Hz) (Figure 7) and a higher frequency of pressure fluctuation in the ventricle and aorta (Figure 8) under the S140G mutation condition. These results are in agreement with findings of Stiles et al. (2008).

The KCNQ1 S140G gain-of-function mutation results in more rapid opening of I_Ks_ channels. Therefore, considerable I_Ks_ current was generated at the early phase of depolarization in endocardium, mid-myocardium, and epicardium cells. This induced rapid repolarization and reduced the APD and ERP. Because APD under the KCNQ1 S140G mutation condition in 1 s BCL is markedly shorter than that under the WT condition (5-fold shorter in the endo-and epicardium and 2.5-fold shorter in the mid-myocardium), alternans occurred at a lower BCL range compared to under the WT condition (Figure 2). These characteristics of restitution due to S140G mutation denote loss of rate-dependent adaptation of APD, which is usually observed in chronic fibrillation patients. In addition, it suggests that ability of cell increases to support high rate electrical excitations, which can be pro-arrhythmic (Kim et al., 2002; Kharche et al., 2012).

The minimum EAT is the starting time of ventricular depolarization, and the maximum EAT is the time at which the entire ventricle is depolarized. The maximum EDT is the time required for repolarization of the entire ventricle. Accordingly, the difference between the maximum and minimum EAT, which is identical to the QRS width, was identical under the WT and S140G mutation conditions. However, the difference between the maximum EDT and minimum EAT, which is equivalent to the QT interval, was, due to the shortened APD, markedly shorter under the S140G mutation condition than under the WT condition (Figure 3C). These results correspond to the clinical findings of Chen et al. (2003).

The shortened APD caused by the KCNQ1 S140G mutation decreased the intracellular Ca^2+^ concentration during the depolarization period by reducing the duration of opening of voltage-dependent L-type Ca^2+^ channels. This reduces Ca^2+^ influx (the Ca^2+^-induced Ca^2+^-released current) from the sarcoplasmic reticulum. The released Ca^2+^ binds to troponin and forms a cross-bridge by structurally modifying tropomyosin. This leads to concentration of the ventricles. However, the reduced Ca^2+^ concentration during the depolarization period caused by the KCNQ1 S140G mutation reduced both active tension and contractile ATP consumption in the ventricles by suppressing cross-bridge formation in myofilaments (Table 1).

The reduced myofilament active tension decreased the ventricular contractile force, which resulted in an increase in LV volume. Accordingly, the stroke volume of the LV was slightly decreased in the S140G mutation condition. However, there was no significant difference. That is, the effect of KCNQ1 S40G mutation on LV pressure was minor in the sinus rhythm despite significant reduction in APD under the S140G mutation condition compared to the WT condition. Therefore, the pressure–volume loop shifted to the right (Figure 4). As the diastolic capacity increased, the ejection fraction, i.e., the ratio of stroke volume to end-diastolic volume, decreased under the S140G mutation condition as compared with the WT condition. In addition, the decreased intracellular Ca^2+^ concentration caused by the KCNQ1 S140G mutation resulted in a slight reduction in work by the left ventricle. However, the amount of ventricular work per unit contractile ATP consumption was increased under the S140G mutation condition (Table 1). In other words, the KCNQ1 S140G mutation increases pumping efficiency. However, the variation in sinus rhythm was neither statistically significant nor clinical important in the KCNQ1 S140G mutation compared to the WT condition. Thus, it is difficult to observe the expression of S140G mutation through specific signs in the normal sinus rhythm.

The shortened APD caused by the KCNQ1 S140G mutation resulted in a short action potential wavelength, and a short action potential wavelength reportedly creates a re-entry wave by including a large number of wavelets (Rensma et al., 1988). Similarly, the reduced cardiac action potential wavelength caused by the KCNQ1 S140G mutation resulted in initiation and maintenance of re-entrant waves in the ventricles under normal electrical conductivity conditions (Figure 5). Therefore, under the KCNQ1 S140G mutation condition, ventricular arrhythmogenesis is facilitated by increased tissue spatial vulnerability.

This study compared cardiac efficiency when the re-entry reached a steady state under the WT and S140G mutation conditions. Under sustained re-entry, the reduced wavelength under the S140G mutation condition increased the rotational rate of re-entry (Figure 6); this led to an elevated action potential firing frequency. In addition, the dominant frequency distribution was wider under the KCNQ1 S140G mutation condition compared to the WT condition (Figure 7). The dominant frequencies were higher and more varied in regions in which the re-entrant rotors remain for a prolonged period than in regions where they do not under both the WT and KCNQ1 S140G mutation conditions (Supplementary Figures 1, 2). These results correspond to those of a prior ventricular fibrillation study using rotor dynamics (Samie et al., 2001).

A high rotational rate of re-entry resulted in an increased rate of fluctuation in LV and aorta pressure under the S140G mutation condition compared to the WT condition (Figure 8A). In addition, during sustained re-entry, the reduced Ca^2+^ concentration caused by the S140G mutation decreased both the formation of cross-bridges in myofilaments and the contractile ATP consumption rate (Figure 8D) by diminishing myofilament active tension. Therefore, the LV pressure–volume loop was shifted to the right under the S140G mutation condition compared to the WT condition (Figure 8B). This reduced the average stroke volume according to Poiseuille's law (Table 1 and Figure 8C). In the presence of re-entry waves, the reduction in the ventricular mechanical pumping function was greater under the KCNQ1 S140G mutation condition than under the WT condition.

Genetic publications have reported gain-of-function pathogenic mutations mainly in three different potassium channels such as KCNQ1, KCNH2, and KCNJ2 (Sarquella-Brugada et al., 2015). Patients with these mutations have shortened atrial and ventricular refractory periods and shortened QT intervals in ECG signal. Short QT syndrome due to these mutation, including KCNQ1 S140G mutation, is an inherited, rare, potential lethal disease characterized by ventricular repolarization alternans, predisposing to atrial fibrillation, syncope, and high incidence of sudden cardiac death. Therefore, the results of this research reflect these clinical results and can be used as a reference for clinical outcomes.

The model in this study has been used for electromechanical computational prediction in several heart failure conditions. Recently, our research group quantitatively predicted the effect of intra-aortic balloon pump function on cardiovascular responses in patients with aortic regurgitation and mitral regurgitation using this electromechanical ventricular model. As such, we successfully computed the reason for the clinical contraindication of intra-aortic balloon pump in patients with aortic regurgitation (Kim et al., 2018). In addition, it is possible to predict the effect of the G229D gene mutation on cardiac performance (Zulfa et al., 2016; Rahma et al., 2018). Therefore, our ventricular model can be used to quantitatively compute the mechanical response in several ventricular assist device conditions as well as in cardiac fibrillation conditions.

There were several limitations to this study that should be addressed. We used “one-way coupling” in which the mechanical stretch do not affect the electrophysiology, although there has been found that mechanoelectric feedback is caused by stretch-activated channels. Second, we considered only the contractile ATP consumption of the myocardium, although ATP is used in other ways, which are sarcoplasmic/endocardium reticulum ATPase (SERCA), plasma membrane Ca^2+^-ATPase circulation. However, there limitations are not expected to greatly alter the main findings of the present study.

## Conclusion

The KCNQ1 S140G mutation, which causes atrial fibrillation, is expressed in ventricular tissue and affects the I_Ks_ current, reducing the APD and ERP. This decreases the conduction wavelength of ventricular tissue, but it also increases cardiac pumping efficiency slightly during sinus pacing. However, the reduced conduction wavelength caused by the S140G mutation increases spatial vulnerability and arrhythmogenesis in ventricular tissue. Furthermore, the short wavelength resulted in many rotors, high rotational rate of rotors, and wide rotor distribution with dominant frequency. Many rotors and their high rotational rates in KCNQ1 S140G mutation can induce difference in systolic timing among ventricular tissue (ventricular dyssynchrony), which can reduce ventricular pumping performance during ventricular fibrillation. In conclusion, we found that although detecting the S140G mutation is difficult in the presence of a normal sinus rhythm, the S140G mutation reduced cardiac mechanical efficiency during sustained re-entry, which increases the risk of cardiac arrest and sudden cardiac death.

## Author contributions

This paper is the intellectual product of the entire team. All of the authors contributed (to varying degrees) to the analytical methods used, research concept, simulation design, simulation source code, simulation process, and writing of the manuscript.

### Conflict of interest statement

The authors declare that the research was conducted in the absence of any commercial or financial relationships that could be construed as a potential conflict of interest.
