# Supplementary material for: Influence of the KCNQ1 S140G Mutation on Human Ventricular Arrhythmogenesis and Pumping Performance: Simulation Study
Source: Front Physiol. 2018 Jul 31;9:926. doi: 10.3389/fphys.2018.00926 (PMC6080549; doi:10.3389/fphys.2018.00926)
Supplement: Supplementary file 1 [file Data_Sheet_1.DOCX]

Supplementary Material

Influence of the KCNQ1 S140G Mutation on Human Ventricular Arrhythmogenesis and Pumping Performance: Simulation Study

Da Un Jeong^1^, Ki Moo Lim^2*^

*** Correspondence:** Ki Moo Lim: kmlimphd@gmail.com

# 3D human ventricular modelling

## Modelling of 3D human ventricles

To compute cardiac tissue and whole-heart electromechanics, we need to solve complex equations, which are based on continuum mechanics and reaction-diffusion system. Numerical solution of these can be derived from frame work of finite element methods (FEM), which requires a reconstruction of heart geometry and fiber structure of the myocardial preparation ([Fijoy et al., 2010](#_ENREF_2)). In order to predict the effect of the KCNQ1 S140G mutation on ventricular fibrillation and cardiac pumping performance, we reconstructed the human ventricular model based on publicly available magnetic resonance (MR) imaging with both fiber orientation information and cardiac tissue heterogeneity information. First, level-set segmentation was performed on the MR image stack to separate myocardium from the suspension media. We used the segmentation function of ITK program for level-set segmentation. The segmentation was achieved through the evolution of a surface Γ, which was implicitly represented as the zero level set of time-dependent 3D function Ф(x, y, z, t).

$\Gamma\left( t \right)=\{x,y,z|Ф\left( x,y,z,t \right)=0\}$ **(1)**

where x, y, z are the Cartesian coordinates, and t is the time. For the implementation of the level set segmentation, the evolution of Ф was as follows:

$Ф=\left\{ \begin{aligned} Ф_{t}=\alpha P\left( x,y,z \right)\left| \nablaФ \right|+\beta k\left( x,y,z \right)|\nablaФ| \\ Ф\left( x,y,z \right), t=0 \end{aligned} \right.$ **(2)**

$P\left( x,y,z \right)=\left\{ \begin{aligned} I\left( x,y,z \right)-L, if I\left( x,y,z \right)<\frac{U-L}{2}+L \\ U-I\left( x,y,z \right), otherwise \end{aligned} \right.$ **(3)**

$k\left( x,y,z \right)=\nabla\cdot(\frac{\nablaФ}{|\nablaФ|})$ **(4)**

where Ф_t_ denotes the partial derivative of Ф with respect to time. α and β are constants whose values are experimentally determined. P(x, y, z) is the propagation term, which consists of the 3D function I(x, y, z) representing the intensity of the 3D image to be segmented. U and L are the upper and lower intensity limits, respectively, which are defined by selecting a region in the input image. k(x, y, z) is the mean curvature term, defined as the divergence of gradient of Ф. This is for smoothing the evolving surface.

Second, the ventricles were detached from the atria. Third, in order to create the atrioventricular boundary, landmark points were manually displayed around ventricles along the atrioventricular border on each tenth slide in the MR image stack. Fourth, a 3D cubic Hermite was aligned along the landmark points of the border, generating a surface of the atrioventricular boundary. The surface mesh was used as a guideline for generating the finite element mesh of the ventricular model.

Fiber and laminar sheet structural information of the ventricles were obtained from the diffusion tensor MR (DTMR) image data set. For incorporate fiber and laminar sheet structure in the ventricular model, tensor and tensor gradients were defined at each node of the finite element mesh and interpolated using Hermite interpolation ([Gurev et al., 2011](#_ENREF_3)). The tensor eigenvectors from the interpolated tensor field denoted the fiber and laminar sheet structured heart, as validated previously ([PATRICK et al., 2005](#_ENREF_4)).

## Modelling of Purkinje network geometry

In the sinus rhythm simulation, we used ventricular mesh including Purkinje fiber to observe the electrical propagation through the fiber. The Purkinje fiber system is a major factor in the synchronization of myocardial activity because of its unique propagation properties and structure ([Berenfeld and Jalife, 1998](#_ENREF_1)). An electrical impulse is generated at the AV node, which is at the top of the Purkinje network, and conduction occurs along the Purkinje network. The Purkinje fibers mesh was established by interconnecting chain of nodes on the Cartesian coordinate system. In order to express the variability of fiber thickness, the fibers were packed parallel to each other. In addition, propagation through the Purkinje network was carried out in consideration of maintaining clinical conduction speed of 200 cm/s. when the electrical impulse reaches the Purkinje terminal nodes at about the same time, it excited the ventricular tissue from the purkinje terminal node, spreading throughout the ventricles.

The procedure that incorporate the Purkinje fiber system was as follows; first, the borders of left ventricular and right ventricular endocardial surfaces are identified on the 3D geometry by edge-detection algorithm and sketched on a 2D bit map. Second, the left and right bundle branches and the Purkinje system were digitized from anatomic data and scaled to fit within border of 2D surfaces. Third, the end points of the Purkinje system were labeled to identify Purkinje muscle junction and connect to the His bundle. Fourth, the 2D bit map digitized Purkinje system was put on the sub-endocardial surface of ventricular model.

# Supplementary Figures and Tables

## Supplementary Figures


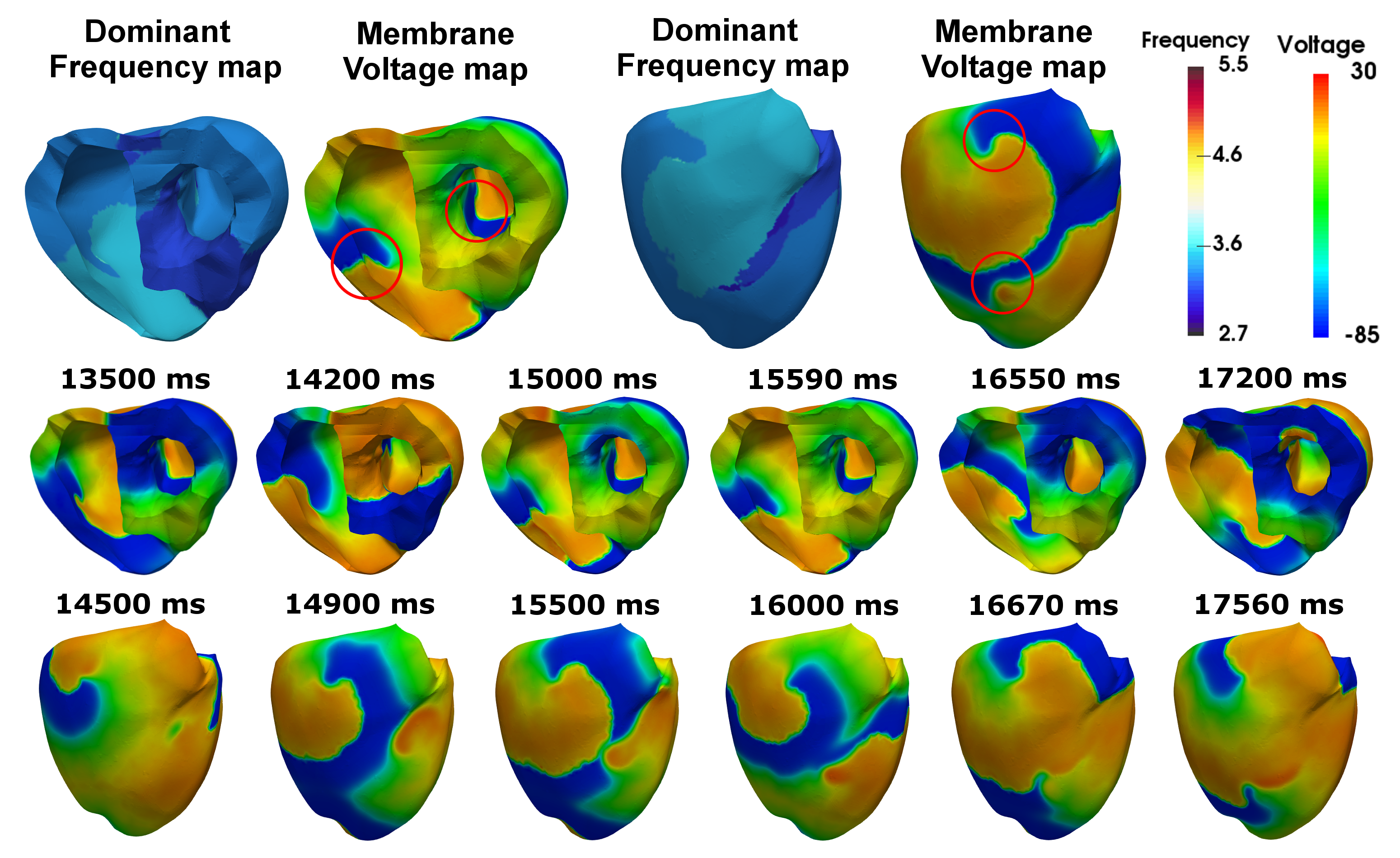


Supplementary Figure 1. Distribution of dominant frequency and membrane voltage map under the WT condition. The membrane voltage map shows the movement of the rotor over time. Red circles denote the center of rotation. WT; wild type.


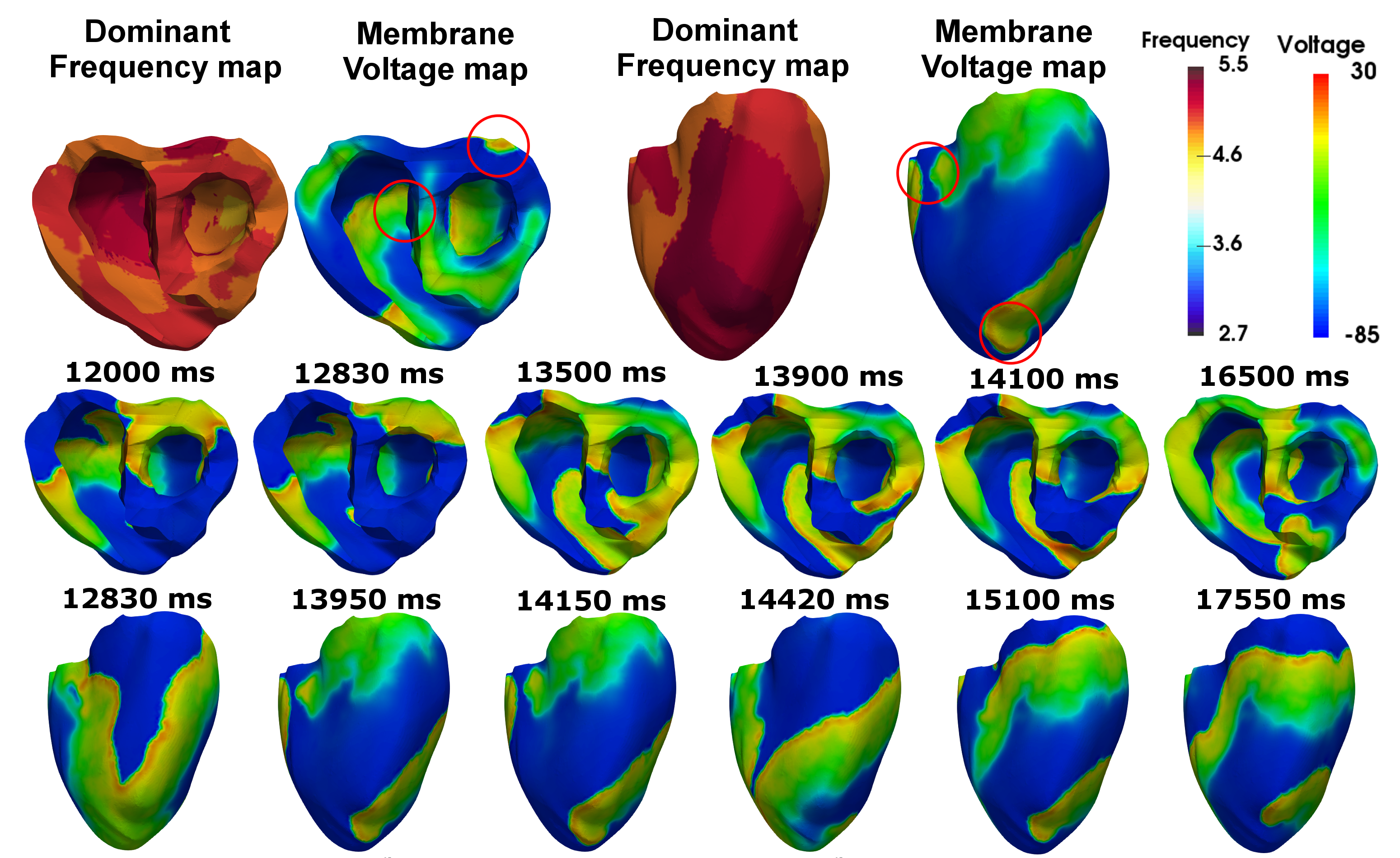


Supplementary Figure 2. Distribution of dominant frequency and membrane voltage map under the S140G mutation condition. The membrane voltage map shows the movement of the rotor over time. Red circles denote the center of rotation.

**Reference**

Berenfeld, O., and Jalife, J. (1998). Purkinje-Muscle Reentry as a Mechanism of Polymorphic Ventricular Arrhythmias in a 3-Dimensional Model of the Ventricles. *Circulation Research* 82(10)**,** 1063-1077. doi: 10.1161/01.res.82.10.1063.

Fijoy, V., Hermenegild, A., J., P.A., Junjie, C., Ferdinand, K., Peter, K., et al. (2010). Image‐based models of cardiac structure in health and disease. *Wiley Interdisciplinary Reviews: Systems Biology and Medicine* 2(4)**,** 489-506. doi: doi:10.1002/wsbm.76.

Gurev, V., Lee, T., Constantino, J., Arevalo, H., and Trayanova, N.A. (2011). Models of cardiac electromechanics based on individual hearts imaging data. *Biomechanics and modeling in mechanobiology* 10(3)**,** 295-306.

PATRICK, H., FAISAL, B.M., I., M.M., and L., W.R. (2005). Measuring and Mapping Cardiac Fiber and Laminar Architecture Using Diffusion Tensor MR Imaging. *Annals of the New York Academy of Sciences* 1047(1)**,** 296-307. doi: doi:10.1196/annals.1341.026.
